# Free ammonia stress rewires microbial signaling networks and constrains metabolic cooperation in oxygenic photogranules

**DOI:** 10.1093/ismejo/wrag225

**Published:** 2026-09-02

**Authors:** Bing Zhang, Xinya Xu, Ming Zhang, Bowen Qi, Hua Ma, Peng Yan, Piet N L Lens, Wenxin Shi

**Affiliations:** Key Laboratory of the Three Gorges Reservoir Region’s Eco-Environment, Ministry of Education, Chongqing University, Chongqing 400045, China; College of Environment and Ecology, Chongqing University, Chongqing 400045, China; Key Laboratory of the Three Gorges Reservoir Region’s Eco-Environment, Ministry of Education, Chongqing University, Chongqing 400045, China; College of Environment and Ecology, Chongqing University, Chongqing 400045, China; Key Laboratory of the Three Gorges Reservoir Region’s Eco-Environment, Ministry of Education, Chongqing University, Chongqing 400045, China; College of Environment and Ecology, Chongqing University, Chongqing 400045, China; Key Laboratory of the Three Gorges Reservoir Region’s Eco-Environment, Ministry of Education, Chongqing University, Chongqing 400045, China; College of Environment and Ecology, Chongqing University, Chongqing 400045, China; Key Laboratory of the Three Gorges Reservoir Region’s Eco-Environment, Ministry of Education, Chongqing University, Chongqing 400045, China; College of Environment and Ecology, Chongqing University, Chongqing 400045, China; Key Laboratory of the Three Gorges Reservoir Region’s Eco-Environment, Ministry of Education, Chongqing University, Chongqing 400045, China; College of Environment and Ecology, Chongqing University, Chongqing 400045, China; Water Supply Sanitation Environmental Engineering, IHE Delft Institute for Water Education, Westvest 7, Delft 2601 DA, The Netherlands; Key Laboratory of the Three Gorges Reservoir Region’s Eco-Environment, Ministry of Education, Chongqing University, Chongqing 400045, China; College of Environment and Ecology, Chongqing University, Chongqing 400045, China

**Keywords:** oxygenic photogranules, free ammonia stress, microbial signaling network, microbial sociobiology, energy allocation, nitrogen removal

## Abstract

Free ammonia (FA) is a prevalent chemical inhibitor in wastewater ecosystems, yet its ecological impacts on microbial communication and cooperation remain poorly understood. Here, we demonstrated that FA stress restructured the community function of oxygenic photogranules (OPGs) by rewiring signaling-associated regulatory networks and redirecting cellular energy allocation. Temporal profiling of extracellular signaling molecules and intracellular regulatory molecules, integrated with metagenomic and metatranscriptomic analyses, revealed a concentration-dependent reconfiguration of microbial signaling. Under low FA exposure (≤1.0 mg/l), diffusible signal factor- and indole-3-acetic acid -associated pathways were more active, coinciding with photogranules consolidation and efficient nitrogen removal. At moderate FA stress (5.0 mg/l), the regulatory landscape shifted toward acyl-homoserine lactone-associated signaling and bis-(3′-5′)-cyclic dimeric guanosine monophosphate-mediated intracellular regulation, consistent with enhanced aggregation and stress adaptation. In contrast, severe FA stress (25.0 mg/l) broadly attenuated signaling-associated pathways, weakened metabolite cross-feeding networks, impaired energy generation, and increased maintenance-related energetic demands. Under these constraints, microbial populations appeared to shift from cooperative metabolism toward self-maintenance-oriented carbon metabolism, evidenced by activation of the carbon-efficient glyoxylate shunt. Collectively, these findings suggest that FA-induced signaling disruption constrains microbial cooperation through cellular energy limitation, highlighting energy allocation as a key determinant of microbial social stability in phototrophic wastewater microbiomes.

## Graphical Abstract

**Graphical Abstract:**
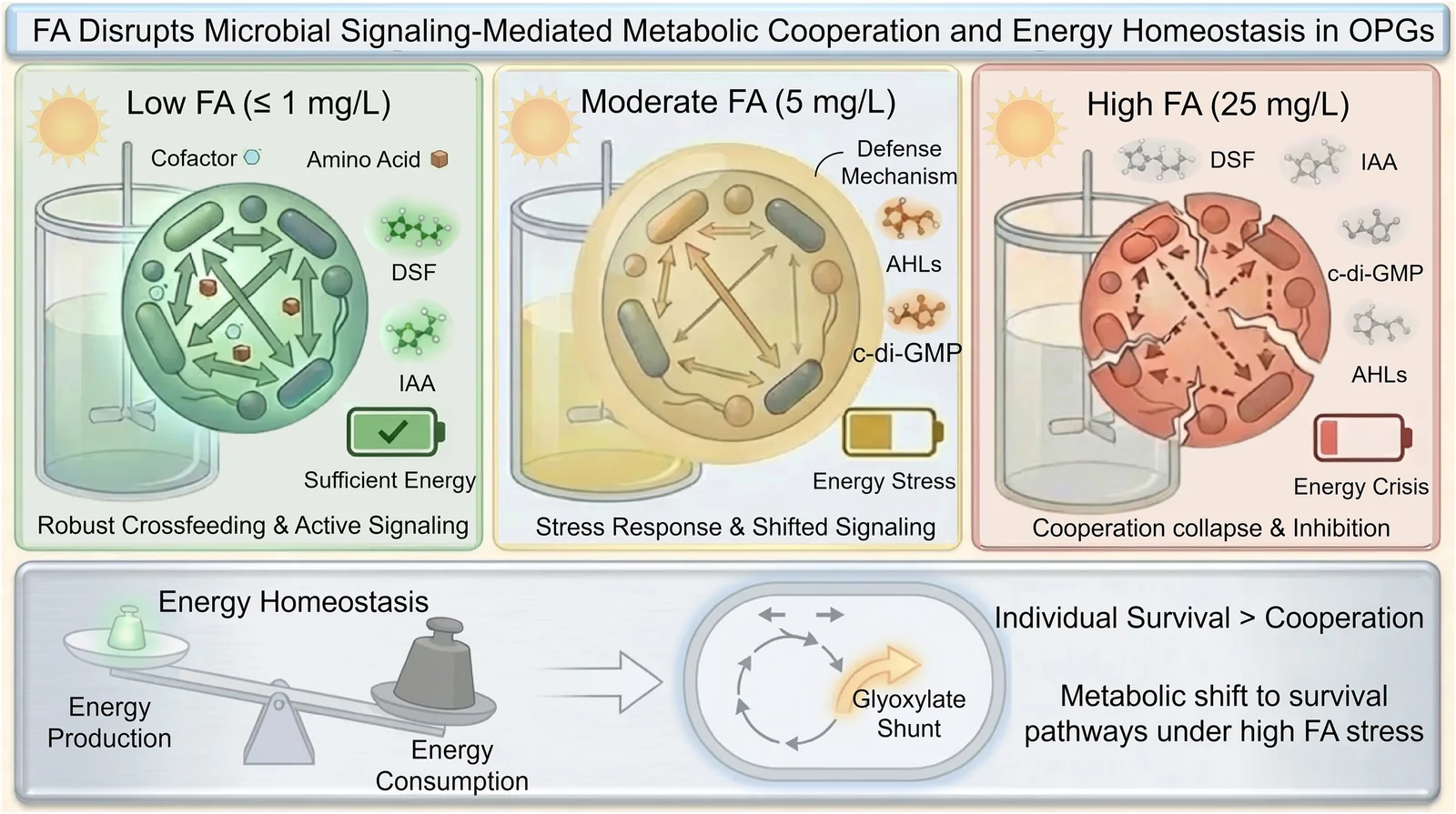

## Introduction

Free ammonia (FA) is a widespread chemical stressor in aquatic and engineered wastewater systems [1]. Its concentration varies widely across wastewater sources, ranging from <1 mg/l in municipal influents to >20 mg/l in alkaline industrial effluents [2]. Although FA serves as a substrate for nitrifiers, elevated concentrations exert inhibitory effects on key microbial guilds involved in biological nitrogen removal [3]. Previous studies have largely interpreted FA toxicity at the level of individual populations, particularly ammonia-oxidizing and nitrite-oxidizing bacteria. However, microbial communities operate as interactive networks in which ecosystem function emerges from coordinated metabolic cooperation, and how FA stress affects these cooperative interactions remains poorly understood.

Oxygenic photogranules (OPGs) provide an ideal model system to investigate stress-induced changes in microbial cooperation. OPGs are self-aggregated consortia in which photosynthetic cyanobacteria supply oxygen and organic metabolites to support bacterial nitrification and heterotrophic metabolism [4]. Bacterial activity, in return, releases nutrients and inorganic carbon to sustain cyanobacterial growth, forming a tightly coupled bacterium-phototroph mutualistic network [5]. Because the stability of this system relies on coordinated metabolic exchanges between distinct functional guilds, environmental stressors such as FA may disrupt not only individual populations, but also the interaction networks that maintain community function.

Microbial signaling provides a key regulatory layer through which microorganisms coordinate collective behaviors and community-level responses. Canonical quorum sensing (QS) signals, such as acyl-homoserine lactones (AHL) [6] and diffusible signal factor (DSF) [7], mediate cell–cell communication and regulate stress tolerance [8], community restructuring [9], and metabolic cooperation [10]. In addition, indole-3-acetic acid (IAA) [11] functions as a microbial signaling molecule involved in bacterium-phototroph interactions and cooperative behaviors. The intracellular second messenger bis-(3′-5′)-cyclic dimeric guanosine monophosphate (c-di-GMP) [12] is not a canonical extracellular QS signal, but it can interact with QS and environmental sensing pathways through regulatory crosstalk, thereby modulating biofilm formation, aggregation, and stress adaptation. Increasing evidence suggests that these signaling and regulatory systems can form integrated networks that modulate community-level responses to environmental stress. In symbiotic microbial systems, these signaling networks can support metabolite cross-feeding, where microorganisms exchange amino acids, vitamins, cofactors, and other public goods to enhance collective performance [10]. However, cooperative metabolism requires sustained energetic investment in signal production, transcriptional regulation, and metabolite biosynthesis. Chemical stress may therefore alter the cost–benefit balance of cooperation by redirecting cellular resources from interpopulation exchange toward self-maintenance, ion homeostasis, and stress resistance. Whether FA stress constrains microbial cooperation by rewiring signaling-associated regulation and redirecting cellular energy allocation remains unclear.

Here, we investigate how FA stress reshapes microbial communication, metabolic cooperation, and cellular energy allocation in OPGs. A sequencing batch reactor (SBR) was operated for 134 days across four FA exposure phases to systematically evaluate concentration-dependent community responses. By integrating temporal profiling of microbial signaling molecules with metagenomic and metatranscriptomic analyses, we aimed to (i) identify FA levels associated with functional destabilization, (ii) characterize the succession and interactions of major signaling and regulatory systems, (iii) evaluate how signaling-associated regulation is linked to metabolite cross-feeding among microbial populations, and (iv) assess whether FA-induced energy constraints contribute to the breakdown of cooperative metabolism. This work provides new ecological insight into how chemical stress reorganizes microbial signaling networks and constrains cooperative behavior in phototrophic wastewater microbiomes.

## Materials and methods

### Reactor operation and experimental design

Seed photogranules were generated by hydrostatically incubating activated sludge under continuous illumination, following a previously published method [13]. Detailed procedures for the seed photogranules preparation are provided in Text S1.

Experiments were conducted in a 1.5-L SBR. A total of 57 hydrostatically cultivated photogranules were used as the inoculum, resulting in an initial mixed liquor suspended solids (MLSS) concentration of approximately 900 mg/l. Illumination was provided by an LED strip (OPPLE, Shanghai, China) mounted above the reactor, supplying photosynthetically active radiation (PAR) of approximately 150 μmol photons/m^2^·s at the reactor surface.

The reactor was operated with a 6-h cycle consisting of alternating dark and light periods of 3 h each. Reactor mixing was maintained at 100 rpm using an overhead stirrer, which was stopped during settling and effluent withdrawal. The mixing speed was selected based on a previously published method [14] to ensure homogeneous mixing and minimizing hydrodynamic shear. No deliberate sludge wasting was performed. The effective sludge retention time was approximately 20 days, governed by the daily withdrawal of approximately 75 ml of mixed liquor for physicochemical and molecular analyses. Synthetic wastewater was fed at the beginning of each cycle under continuous mixing, followed by reaction, biomass settling, and effluent withdrawal. Detailed operational parameters are provided in Text S2.

To evaluate the microbial responses of OPGs to increasing FA stress, the reactor was operated continuously for 134 days under four sequential FA exposure phases: 0.1 mg/l in Phase I (days 1–34), 1.0 mg/l in Phase II (days 35–66), 5.0 mg/l in Phase III (days 67–98), and 25.0 mg/l in Phase IV (days 99–134). The FA gradient was selected based on FA concentrations reported in domestic wastewater (0.14–1.38 mg/l) and FA levels potentially encountered in industrial wastewater or high-strength nitrogen treatment systems (5–50 mg/l) [15].

### Synthetic wastewater and FA regulation

Synthetic domestic wastewater was used as the influent for the SBR. It contained sodium acetate (4.68 mM) as the sole organic carbon source, together with essential nutrients (0.20 mM KH_2_PO_4_, 0.10 mM CaCl_2_, 0.20 mM MgSO_4_·7H_2_O, and 0.04 mM FeSO_4_·7H_2_O) and 1 ml/l trace element solution. The detailed composition of the influent medium is provided in Text S2.

The FA concentration was calculated as a function of NH_4_^+^-N concentration, pH, and temperature according to Eq. (1) [16]:


(1) \begin{align*} \kern0.5em \mathrm{FA}\ \left(\mathrm{mg}/\mathrm{l}\right)=\frac{17\times C\left(\mathrm{NH}{4}^{+}-\mathrm{N}\right)\times{10}^{\mathrm{pH}}}{14\times \left(\frac{{\mathrm{e}}^{6344}}{273+T}+{10}^{\mathrm{pH}} \right)} \end{align*}

where $C\left(\mathrm{NH}{4}^{+}-\mathrm{N}\right)$ is the ammonium nitrogen concentration (mg/l), pH is the bulk liquid pH, and *T* is the temperature (°C).

Target FA levels were established indirectly by adjusting the ammonium loading and alkalinity. NH_4_Cl was supplemented as the sole nitrogen source, maintaining total ammonia nitrogen (TAN) concentrations of approximately 25, 50, 100, and 150 mg N/l in Phases I–IV, respectively. NaHCO_3_ was added to provide alkalinity and stabilize pH. Because FA exposure in the reactor depends on the in-reactor TAN, pH, and temperature, FA concentrations were calculated using the measured bulk-liquid pH. During the experiment, the bulk-liquid pH was monitored daily, with measured ranges of 6.9–7.2 in Phase I, 7.3–7.6 in Phase II, 7.7–7.9 in Phase III, and 8.3–8.5 in Phase IV. The reactor was operated at a controlled temperature of 23 ± 3°C.

### Metagenomic and metatranscriptomic sequencing

Total genomic DNA and RNA were extracted from biomass samples collected across the four operational phases using the FastDNA SPIN Kit for Soil (MP Biomedicals, Solon, OH, USA) and the E.Z.N.A. Soil RNA Kit (Omega Bio-tek, Norcross, GA, USA), respectively, following the manufacturers’ protocols. For metagenomic analysis, DNA was fragmented and used to construct paired-end sequencing libraries, which were sequenced on a HiSeq 4000 System (Illumina Inc., San Diego, CA, USA). For metatranscriptomic analysis, total RNA was subjected to quality control, followed by ribosomal RNA removal. The resulting mRNA-enriched samples were used to construct cDNA libraries on a HiSeq 2500 System (Illumina Inc., San Diego, CA, USA).

### Bioinformatic analysis and genome binning

Metagenomic reads were quality-filtered and assembled prior to genome binning. Metagenome-assembled genomes (MAGs) were reconstructed using a binning-based workflow, followed by phylogenetic classification and quality assessment [17]. MAG quality was evaluated based on genome completeness and contamination metrics. Open reading frames (ORFs) were predicted from assembled contigs and functionally annotated using KEGG databases. Metatranscriptomic reads were mapped to predicted genes to quantify transcriptional activity. Gene expression levels were normalized and quantified as fragments per kilobase of transcript per million mapped reads (FPKM) [18]. Differential gene expression among experimental phases was evaluated using Fisher’s exact test [19]. All raw sequencing data have been deposited in the NCBI Sequence Read Archive (SRA) under BioProject accession number PRJNA1403730.

### Identification of genes associated with signaling-related transcriptional regulators

Clp and LuxR are transcriptional regulators associated with DSF- and AHL-mediated signaling pathways, respectively [20, 21]. To identify putative target genes regulated by these transcriptional regulators, upstream promoter regions of predicted ORFs were screened for conserved binding motifs using a custom Python script [22]. Promoter regions were defined as the 500 bp sequences upstream of each ORF. The conserved Clp-binding motif (TGTGA-XXXXXX-TCACA) was identified using a minimum identity threshold of 70.0%, whereas the invariant LuxR-binding motif (CT-XXXXXXXXXXXX-AG) required 100.0% sequence identity [23]. When multiple candidate binding sites were detected within a promoter region, only the highest-scoring site was retained for subsequent analysis.

### Chemical and microbial analyses

Concentrations of NH_4_^+^-N, NO_2_^−^-N, NO_3_^−^-N, total nitrogen (TN), as well as sludge volume index at 5 and 30 min (SVI_5_ and SVI_30_) and MLSS, were determined following the standard methods [24]. Photosynthetic pigments, including chlorophyll a, chlorophyll b, chlorophyll c, and phycobilin, were quantified following a modified protocol [4]. The morphology and microstructure of OPGs were examined using an optical microscope (Olympus, BX60, Tokyo, Japan) and a scanning electron microscope (SEM, GeminiSEM 300, ZEISS, Oberkochen, Germany), respectively. The mean particle size and size distribution of OPGs were measured using a laser particle size analyzer (Malvern Panalytical, Mastersizer 3000, Worcestershire, UK). Procedures for extracellular polymeric substances (EPS) extraction and characterization are described in Text S3. Microbial nitrogen metabolism and electron transport system activity (ETSA) were measured according to previously described procedures [25] and are summarized in Text S5. The adenosine triphosphate (ATP) content in OPGs was extracted and quantified according to previous studies [26] and the protocol of an ATP assay kit (S0026, Beyotime, China).

### Quantification of microbial signaling and regulatory molecules

Methods for the extraction and quantification of microbial signaling and regulatory molecules, including AHL, c-di-GMP, IAA, and DSF, are provided in Text S4.

### Statistical analysis

Structural equation modeling was constructed to quantify the total, direct, and indirect effects of FA on microbial signaling and regulatory molecules, including AHL, c-di-GMP, IAA, and DSF. Model fit was evaluated using the chi-square test and the root-mean-square error of approximation (RMSEA) [27]. Path coefficients were calculated using the maximum likelihood estimation method in R software (version 4.5.1). Analysis of covariance (ANCOVA) was performed using FA phase as the fixed factor and operational time as a covariate to assess potential temporal effects. Differences were considered statistically significant at *P* < .05 (Tables S2–S8).

All statistical analyses were performed using SPSS 27.0 unless otherwise stated. Prior to statistical testing, data were assessed for normality and homogeneity of variance. Differences among experimental phases were evaluated using one-way analysis of variance (ANOVA), with significance defined as *P* < .05.

## Results

### FA stress destabilizes photogranule structure and nitrogen removal performance at elevated concentrations

Increasing FA concentrations induced pronounced phase-dependent changes in both the structural stability of OPGs and nitrogen removal performance. During Phases I and II (0.1–1.0 mg/l FA), photogranule development was markedly enhanced. The mean particle diameter increased to 1714.21 μm, accompanied by improved settling performance, as indicated by an SVI_5_ of 41.53 ml/g (Fig. 1A–E). Concurrently, EPS content increased from 58.31 to 91.42 mg/g MLSS, with the protein-to-polysaccharide ratio (PN/PS) rising from 1.75 to 2.80, suggesting the development of a protein-enriched EPS matrix (Fig. 1C). Photosynthetic pigments also accumulated steadily, with phycobilin and chlorophyll reaching 0.05 mg/l and 23.53 mg/l, respectively (Fig. 1D and E), indicating strengthened phototrophic activity during early granulation. Microscopic observations further confirmed the morphological transition from irregular aggregates in Phase I to compact spherical structures in Phase II (Figs S1 and S2), consistent with progressive structural consolidation.

**Figure 1:**
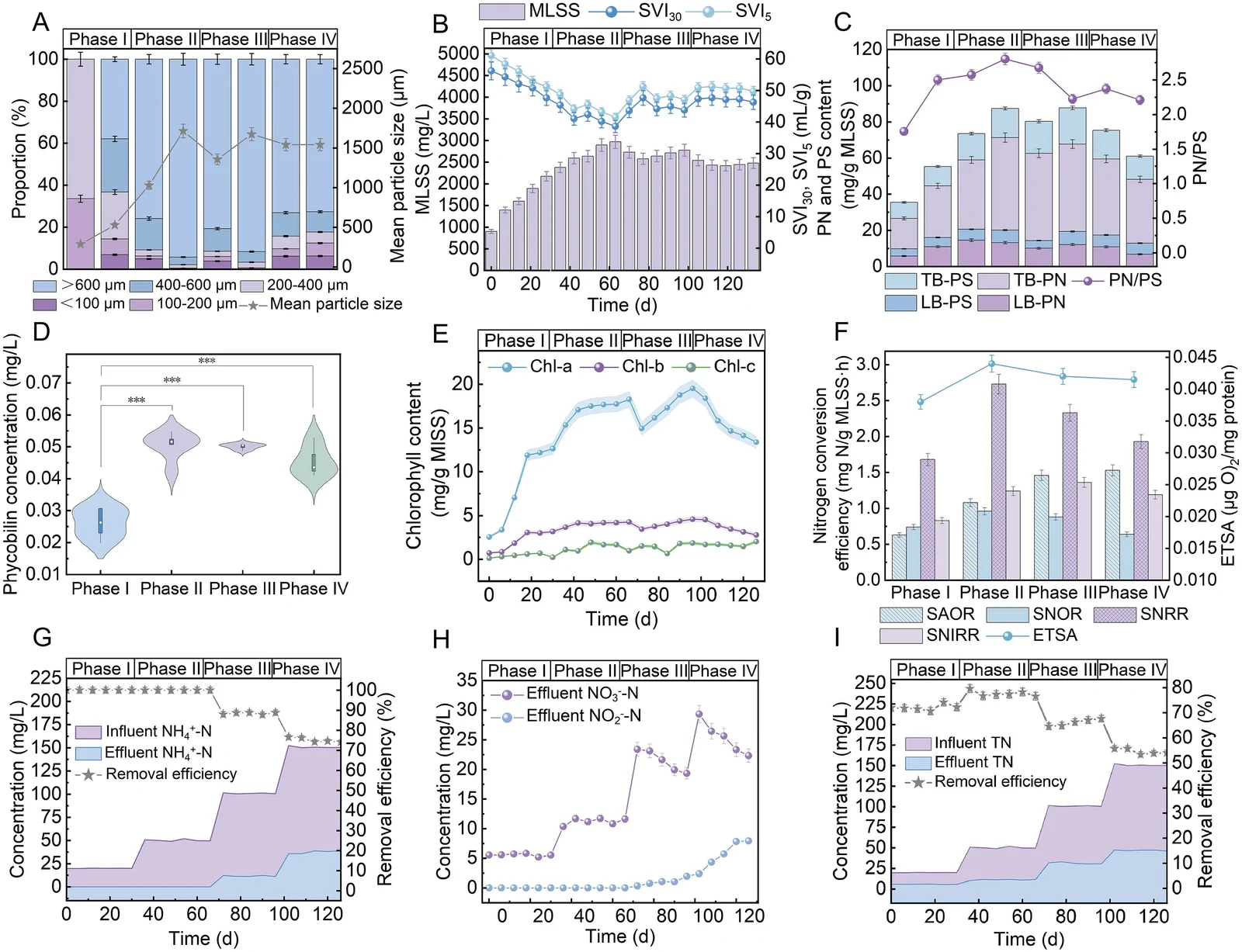
Physicochemical characteristics of OPGs and reactor performance during Phases I–IV. (A) Particle size distribution and D(4,3) volume-weighted mean particle diameter of OPGs; the star symbols and connecting line indicate the D(4,3) mean diameter. (B) MLSS, SVI_30_ and SVI_5_. (C) EPS content. (D) Phycobilin concentration. (E) Chlorophyll concentration. (F) Nitrogen transformation activities and ETSA, including SAOR, SNOR, SNIRR, SNRR, and ETSA. (G) Ammonium removal performance. (H) Effluent NO_3_^−^-N and NO_2_^−^-N concentrations. (I) TN removal performance.

At moderate FA levels (Phase III, 5.0 mg/l), the physicochemical properties of OPGs became more variable. SVI_5_, MLSS, and EPS content initially declined but partially recovered by the end of this phase, reaching 48.34 ml/g, 2864.06 mg/l, and 88.76 mg/g MLSS, respectively (Fig. 1A–E). The mean particle diameter remained relatively stable at 1712.68 μm, whereas phycobilin and chlorophyll concentrations increased by 2.2% and 14.0%, respectively, compared with Phase II (Fig. 1D and E). These responses indicate partial physiological adaptation and structural resilience of the community under moderate FA stress.

In contrast, exposure to the highest FA concentration in Phase IV (25.0 mg/l) led to pronounced structural destabilization. Although the mean particle diameter and SVI_5_ remained at 1541.18 μm and 50.78 ml/g, respectively, within the typical range for granular sludge (Fig. 1A and B), multiple indicators pointed to deteriorating structural integrity. These included visible fissures and voids under SEM observation (Fig. S2), a marked shift in particle size distribution toward fragmentation (characterized by a decrease in >600 μm granules and an increase in <100 μm fines, Fig. 1A), and a decline in EPS content to 60.28 mg/g MLSS (Fig. 1C). Concurrently, photosynthetic pigment concentrations decreased from their peak levels in Phase III to 0.04 mg/l for phycobilin and 17.34 mg/l for chlorophyll in Phase IV, indicating a reversal of the previous upward trend and suppression of phototrophic activity under severe FA stress (Fig. 1D and E).

These structural changes were accompanied by clear functional responses in nitrogen transformation. During the low-FA stages (Phases I and II, 0.1–1.0 mg/l), ammonium removal remained complete, with TN removal increasing from 72.3% to 80.4%, paralleling the progressive stabilization of photogranule structure (Fig. 1G–I). Phase III (5.0 mg/l FA) represented a critical functional transition, characterized by substantial nitrite accumulation and significant declines in both ammonium and TN removal efficiencies (*P* < .05), indicating the onset of FA-induced disruption of nitrogen transformation. Although partial recovery occurred toward the end of this phase, ammonium and TN removal efficiencies recovered only to 88.8% and 67.6%, respectively, remaining below the low-FA levels (Fig. 1G–I). Under the highest FA stress in Phase IV, continuous accumulation of both nitrite and nitrate was observed, accompanied by a further decline in TN removal efficiency to 55.0% (Fig. 1I).

Microbial activity assays provided additional mechanistic insight into these performance shifts. The specific ammonia oxidation rate (SAOR) increased progressively with FA concentration, reaching a maximum of 1.53 mg N/(g MLSS·h) in Phase IV (Fig. 1F), suggesting that ammonia-oxidizing potential was retained under assay conditions. In contrast, the specific nitrite oxidation rate (SNOR) peaked at 0.96 mg N/(g MLSS·h) in Phase II and declined thereafter, consistent with the observed nitrite accumulation (Fig. 1F). Similarly, specific nitrite and nitrate reduction rates (SNRR and SNIRR) decreased after Phase II, reaching final values of 1.25 and 1.93 mg N/(g MLSS·h), respectively, in Phase IV (Fig. 1F). ETSA and intracellular ATP levels increased during Phases I and II but declined markedly during Phases III and IV (Fig. S3), suggesting progressive impairment of cellular energy metabolism under elevated FA stress. These phase-dependent differences remained significant after controlling for operation time using ANCOVA (*P* < .05; Tables S2–S8), suggesting that FA exposure phase, rather than operational time alone, was the major driver of the observed changes. Collectively, these results indicate that low FA exposure promoted photogranule development, whereas elevated FA stress progressively weakened photogranule structure and impaired nitrogen transformation pathways.

### FA stress rewires microbial signaling and regulatory networks

To determine whether FA stress altered microbial signaling and regulatory interactions, we quantified the temporal dynamics of four major microbial signaling or regulatory molecules, including AHL, c-di-GMP, IAA, and DSF, across the operational phases.

AHL and c-di-GMP increased markedly during Phases I and II, rising by 156.3% and 234.7%, respectively (Fig. 2A and B). Both continued to increase in Phase III, reaching peak levels 1.34- and 1.28-fold higher than those in Phase II, respectively, before declining sharply in Phase IV (Fig. 2A and B). In contrast, DSF and IAA reached their maximum levels earlier in Phase II, and decreased progressively thereafter (Fig. 2B). These divergent temporal patterns indicate that extracellular signaling molecules and intracellular regulatory messengers exhibited distinct sensitivities to increasing FA exposure.

**Figure 2:**
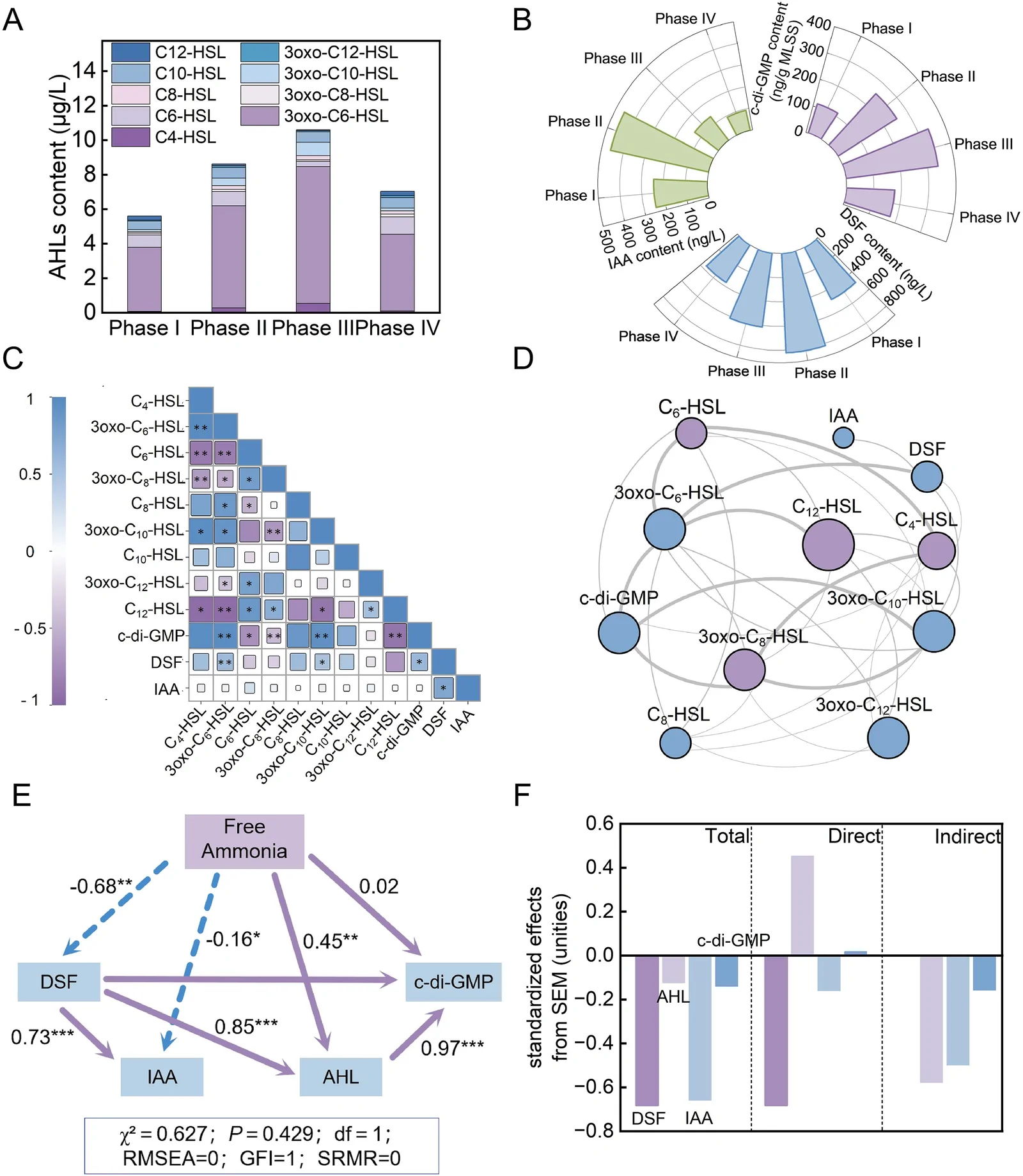
Profiles of microbial signaling molecules and their regulatory relationships under different FA exposure phases. Concentration of AHLs (A), c-di-GMP, IAA, and DSF (B) in four phases. (C) Correlations among signal molecules in different phases. (D) Significant (*P* < .05) and strong (Spearman’s *R* > 0.7) connections between signal molecules were selected and visualized. The node size is proportional to the number of connections, and the edge thickness implies the relevance degree. (E and F) Structural equation model based on the total, direct, and indirect effects of FA on AHLs, c-di-GMP, IAA as well as DSF. Numbers adjacent to arrows are standardized path coefficients and indicate the effect size of the relationship. Asterisks (^*^) indicate the significant differences: ^*^, *P* < .05; ^**^, *P* < .01; ^***^, *P* < .001.

Despite these phase-dependent shifts, significant correlations among signaling-related molecules were consistently detected. Within the AHL family, C_4_-HSL was significantly correlated with 3-oxo-C_6_-HSL (ρ = 0.82, *P* < .05; Fig. 2C and D). Cross-pathway associations were also evident, including significant correlations between 3-oxo-C_10_-HSL and c-di-GMP (ρ = 0.85, *P* < .05), and between DSF and IAA (ρ = 0.51, *P* < .05). These relationships suggest potential coordination among heterogeneous signaling and regulatory systems during photogranule development and stress adaptation (Fig. 2C and D).

FA concentration was closely associated with these signaling dynamics. At low FA levels (0.1–1.0 mg/l), FA showed positive correlations with most microbial signaling-associated molecules, including C_4_-HSL (ρ = 0.65, *P* < .05) and 3-oxo-C_10_-HSL (ρ = 0.58, *P* < .05) (Fig. S4). However, when FA concentrations exceeded 5.0 mg/l, this trend was reversed, with the signaling abundance declining markedly as FA levels increased further. C-di-GMP (ρ = −0.87, *P* < .05) and DSF (ρ = −0.91, *P* < .05) exhibited strong negative correlations with FA concentration under elevated FA exposure (Fig. S4). These patterns indicate a biphasic response in which low FA exposure was associated with enhanced signaling activity, whereas severe FA stress broadly suppressed signaling and regulatory molecules.

To further resolve regulatory interactions among signaling pathways, a structural equation model was constructed. The model showed excellent goodness-of-fit (χ^2^ = 0.627, *P* = 0.429, df = 1, RMSEA = 0.000, GFI = 1, and SRMR = 0). FA showed significant direct associations with AHL (estimate = 0.45, *P* < .05), IAA (estimate = −0.16, *P* < .05), and DSF (estimate = −0.68, *P* < .05) (Fig. 2E and F). Indirect associations were also detected, with FA being linked to IAA (estimate = 0.73, *P* < .05) and AHL (estimate = 0.85, *P* < .05) through DSF-mediated pathways, and to c-di-GMP (estimate = 0.97, *P* < .05) through AHL-associated pathways (Fig. 2E and F). Together, these results indicate that FA stress reshaped microbial signaling not only by altering the abundance of individual signaling-related molecules but also by reorganizing the interaction network among signaling pathways.

### Genetic potential and transcriptional reprogramming of signaling-associated pathways

To investigate the genetic basis underlying FA-induced changes in microbial signaling and regulation, MAGs harboring key bacterial communication genes (BCGs) and signaling-associated regulatory genes were reconstructed. A total of 10 medium- to high-quality MAGs (completeness >65.0%, contamination <10.0%) were recovered from genome binning (Tables 1 and S1). These MAGs were affiliated mainly with *Pseudomonadota* (PSE), *Bacteroidota* (BAC), *Acidobacteriota* (ACI), and *Cyanobacteria* (CYA) (Fig. 3A). Together, these populations accounted for 41.7%–54.2% of the community across all operational phases (Table 1). Among them, CYA1 was the most abundant phototrophic population, with relative abundances ranging from 16.1% to 21.4% (Table 1).

**Table 1:** Relative abundance of the 10 recovered MAGs (identified by key BCGs related to AHLs, c-di-GMP, IAA, and DSF) across the four experimental phases in OPG systems.

**MAG** **ID**	**Relative abundance (%) in phase I**	**Relative abundance (%) in phase II**	**Relative abundance (%) in phase III**	**Relative abundance (%) in phase IV**
PSE1	0.8	2.0	3.1	6.8
PSE2	3.9	3.8	2.6	1.6
PSE3	2.1	1.8	1.9	1.2
PSE4	1.9	2.8	1.9	1.6
PSE5	2.2	3.9	1.9	0.7
BAC1	1.3	2.9	2.3	1.4
BAC2	1.9	3.5	2.4	1.1
CYA1	16.1	21.4	19.6	17.9
CYA2	9.8	10.3	8.1	9.0
ACI	1.9	1.8	1.2	0.5

**Figure 3:**
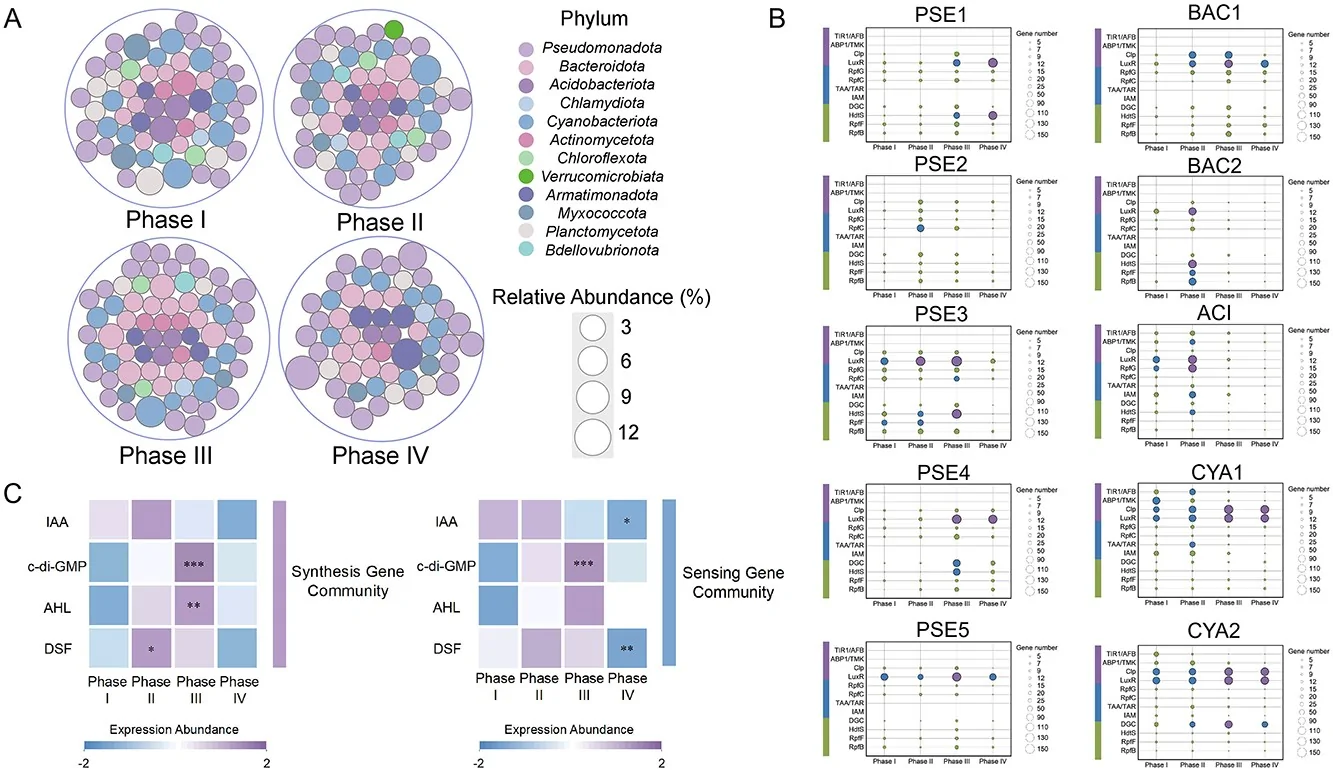
Microbial community composition, signaling-associated gene abundance, and pathway expression across different phases. (A) Relative abundance of MAGs at the phylum level. (B) Numbers of genes encoding signaling-associated pathways in MAGs. (C) Expression of BCGs and signal pathways at each phase. The asterisk represents the significant difference from Phase I. Asterisks (^*^) indicate the significant differences with Phase I: ^*^, *P* < .05; ^**^, *P* < .01; ^***^, *P* < .001.

Genes associated with AHL and DSF synthesis were widely distributed across the recovered MAGs. Bacterial MAGs affiliated with PSE, BAC, and ACI commonly harbored genes encoding AHL synthases (e.g. HdtS), alongside DSF biosynthesis and regulatory components, including RpfF/RpfG (Fig. 3B). These patterns suggest a broad genetic potential for extracellular signaling and community-level coordination among bacterial populations. Multiple genes encoding diguanylate cyclases (DGCs), which are responsible for c-di-GMP synthesis, were also detected, indicating that c-di-GMP-mediated intracellular regulation was widely distributed across the recovered MAGs. The widespread occurrence of DGC-encoding genes suggests that c-di-GMP may serve as an important regulatory hub, potentially coordinating intracellular regulation with DSF-dependent extracellular signaling [28]. In contrast, genes related to IAA biosynthesis, including TAA/TAR and IAM, were detected at relatively low copy numbers in cyanobacterial MAGs (CYA1 and CYA2) and a subset of ACI MAGs, suggesting a more restricted genetic potential for IAA-associated interactions within the bacterial-phototrophic consortium (Fig. 3B).

Although the genetic repertoire remained largely stable across phases, the transcriptional activity of signaling-associated pathways shifted markedly with increasing FA stress (Fig. 3C). During Phases I and II (0.1–1.0 mg/l FA), genes involved in DSF and IAA biosynthesis and sensing were significantly upregulated (*P* < .05), indicating that these pathways were transcriptionally active under low FA exposure (Fig. 3C). At moderate FA exposure (Phase III, 5.0 mg/l), transcriptional activity shifted toward c-di-GMP and AHL-mediated pathways, as reflected by the significantly elevated expression of genes encoding synthases, receptors, or regulatory components (*P* < .05) (Fig. 3C). This shift indicates a reprogramming of regulatory strategies from early-stage growth-associated signaling toward pathways associated with aggregation, biofilm stability, and stress adaptation.

At the highest FA concentration (Phase IV, 25.0 mg/l), the transcription of synthase, receptor, and regulatory genes associated with all four signaling pathways declined markedly, indicating global suppression of signaling-associated activity (Fig. 3C). This repression occurred despite the continued presence of these genes within MAGs, suggesting that severe FA stress constrained microbial signaling networks primarily at the transcriptional regulatory level rather than through a loss of genetic potential.

Collectively, these results reveal a stepwise reconfiguration of signaling strategies in OPGs under FA stress, shifting from DSF/IAA-dominated signaling at low FA concentrations, through c-di-GMP/AHL-dominated regulation at moderate FA exposure, to a broad transcriptional attenuation under severe FA stress.

### Signaling-associated metabolite cross-feeding supports cooperative nitrogen metabolism

Metatranscriptomic analysis suggested extensive potential metabolic interactions among community members, particularly through metabolite cross-feeding. Genes involved in metabolite biosynthesis were broadly upregulated during Phases I–III but showed pronounced downregulation in Phase IV (Figs S5 and S7), indicating that cooperative metabolic exchange was weakened under severe FA stress. Six MAGs, including PSE1, PSE3, PSE4, PSE5, CYA1, and CYA2, exhibited consistently high transcriptional investment in biosynthetic pathways and were therefore identified as major potential contributors to cross-feeding interactions. In contrast, the remaining MAGs appeared to contribute less to cross-feeding due to either low transcriptional activity or the absence of key biosynthetic functions.

Amino acid exchange emerged as a central cooperative strategy. PSE1 and CYA1 showed high expression of genes involved in the biosynthesis of energetically expensive amino acids such as tryptophan and lysine, due to complex biosynthetic pathways and high ATP requirements. In contrast, PSE4 and CYA2 preferentially expressed pathways for less costly amino acids including glycine, arginine, and proline, which are associated with shorter biosynthetic routes, abundant precursors, and lower energetic costs (Fig. 4). Protein degradation may have provided an additional source of amino acids [29]. PSE2 showed the strongest proteolytic potential, as evidenced by the highest abundance of peptidase genes, with a gene copy number of 14 (Fig. S6), suggesting that it may contribute to amino acid availability through protein degradation. These exchanged amino acids may have further supported the biosynthesis of essential cofactors. For instance, elevated expression of riboflavin (VB2) and thiamine (VB1) biosynthesis genes in PSE3 and PSE5 may have been supported by amino acids potentially supplied by PSE1 and CYA2, as indicated by the elevated expression of corresponding cofactor biosynthetic genes (Fig. S7). These patterns suggest tightly coordinated metabolic interactions within the community.

**Figure 4:**
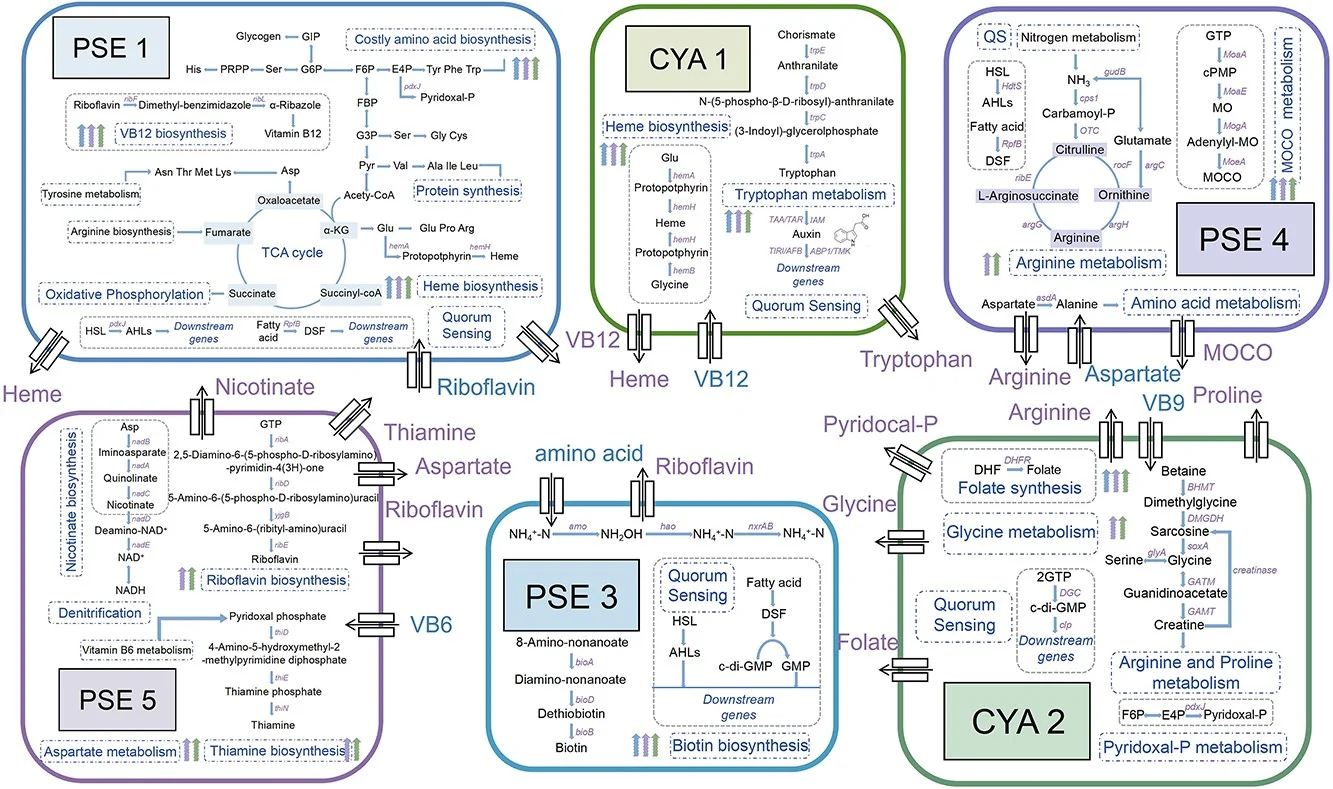
Schematic profiles of cross-feeding among PSE1, PSE3, PSE4, PSE5, CYA1, and CYA2 under FA stress. Blue, purple, and green arrows represent up-regulation of the pathway in Phase II, Phase III, and Phase IV, respectively.

Evidence further suggested that these cooperative interactions were linked to signaling-associated regulation. Genes encoding Clp and LuxR, key transcriptional regulators in DSF- and AHL-mediated pathways, were detected in PSE1, PSE4, CYA1, and CYA2 (Fig. S8). Promoter analyses identified Clp- and LuxR-binding motifs upstream of multiple metabolic genes, supporting a potential regulatory link between signaling pathways and metabolic gene expression. Consistently, the transcription levels of these metabolic genes were correlated with signaling pathway activity. For instance, AHL-associated activity in PSE1 was positively correlated with genes involved in the biosynthesis of tryptophan, tyrosine, glutamine, and arginine, whereas DSF-related activity in CYA1 coincided with elevated transcription of tryptophan- and glutamate-related pathways (Fig. S8). These patterns suggest that signaling-associated transcriptional regulation may contribute to the coordination of metabolites exchange.

At the community level, these cross-feeding interactions were connected to nitrogen transformation processes [30]. Functional profiles indicated that PSE1-PSE4 contributed primarily to nitrification, whereas PSE5, BAC1, and BAC2 contributed to denitrification (Fig. S9). The availability of exchanged amino acids and cofactors likely supported the activity of these functional populations. For instance, aspartate and nicotinate supplied to PSE5 could promote NADH generation, thereby providing reducing power for denitrification and contributing to nitrogen removal under low to moderate FA exposure (Fig. 4).

Under elevated FA stress, signaling activity was severely weakened and cooperative metabolic networks were disrupted. At high FA concentrations, particularly in Phase IV, several populations reduced the transcription of genes involved in shared metabolite production. BAC2 exemplified this shift by silencing the expression of genes responsible for synthesizing public goods such as MOCO, VB12, thiamine, glutamate, and aspartate (Fig. S7). Metabolic division of labor may enable populations to reduce individual energy burdens through specialization [31]. For instance, PSE1 and CYA1 preferentially invested in the biosynthesis of energy-intensive amino acids, whereas other populations may have utilized these shared metabolites to reduce their own biosynthetic investment. When severe FA stress suppressed shared metabolite production, this energetic advantage was likely weakened [32]. As a result, community members may have required greater autonomous biosynthetic investment or alternative uptake strategies, potentially increasing biosynthetic energy demands. These changes may have exacerbated metabolite-availability imbalances and increased cellular energetic constraints, thereby compromising stress tolerance and contributing to the deterioration of nitrogen removal performance. (Figures S7, S3, and  S9).

### Elevated FA stress disrupts energy homeostasis and drives metabolic reallocation

The weakening of cooperative metabolic interactions was accompanied by pronounced disturbances in cellular energy metabolism. Intracellular ATP levels increased during Phases I and II, reaching 3.68 mg/g MLSS, but declined sharply under elevated FA stress, falling to 2.04 mg/g MLSS in Phase IV (Fig. S3).

Physiologically, FA (NH_3_) can readily diffuse across cell membranes and be rapidly protonated to NH_4_^+^, thus disturbing intracellular pH homeostasis, inducing potassium loss and triggering osmotic stress [33]. Consistent with this stress response, genes encoding K^+^ influx transporters, including Trk and Kup, were strongly upregulated by 296.2% in Phase II and 77.0% in Phase III, but declined markedly by 79.2% in Phase IV (Fig. S10). In contrast, K^+^ efflux systems remained transcriptionally active even under the highest FA concentration. Expression of the Na^+^/H^+^ antiporter NhaBC, a key component involved in sodium and pH homeostasis, also increased by 3.70-fold, 1.76-fold, and 1.21-fold, respectively, in Phases II–IV (Fig. S10), indicating sustained activation of ion-regulatory mechanisms under FA stress [34].

Energy generation pathways exhibited coordinated changes across operational phases. During Phases I and II, both photosynthetic photophosphorylation and oxidative phosphorylation were enhanced. For instance, expression of *psaC*, encoding a photosystem I component, increased by 2.20-fold, and the NADH dehydrogenase gene *ndhH* increased by 1.77-fold, accompanied by a 1.19-fold increase in ATP synthase expression (Fig. 5A and B). This coordinated enhancement likely supported efficient ATP production under low FA exposure.

**Figure 5:**
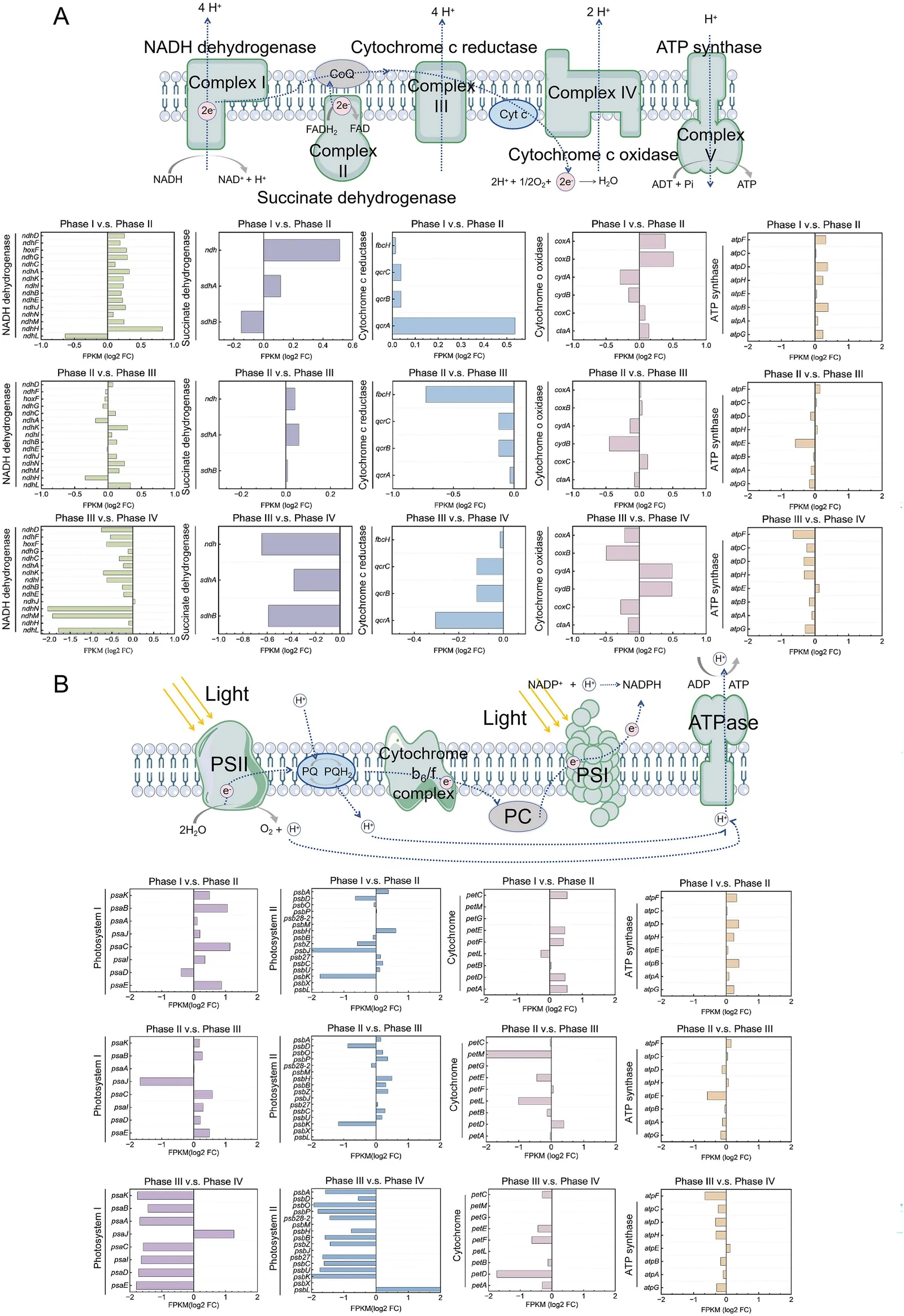
Relative expression of genes involved in energy metabolism across different phases. (A) Relative expression levels of genes encoding oxidative phosphorylation. (B) Relative expression levels of genes encoding photosynthetic photophosphorylation in Phases I–IV.

At moderate FA levels (5.0 mg/l, Phase III), the bioenergetic coupling became less stable. Although photosynthetic genes remained partially active, transcription of cytochrome b_6_f declined slightly by 2.8%, whereas Complexes III and IV of oxidative phosphorylation declined by 22.6% and 0.4%, respectively (Fig. 5A and B). Concurrently, ATP synthase expression decreased relative to Phase II, suggesting a coordinated downregulation of the oxidative phosphorylation machinery under moderate FA stress.

As FA levels increased to 25 mg/l (Phase IV), repression of energy metabolism became more pronounced. Transcripts encoding photosystems I and II components declined by 66.7% and 74.8%, respectively, with components of the respiratory chain decreasing by up to 30.8% (Fig. 5A and B). These changes were consistent with the marked decline in ATP synthase expression and intracellular ATP levels, indicating impaired energy generation under severe FA stress.

Central carbon metabolism exhibited a similar reorganization under increasing FA stress. During Phases I and II, genes associated with the tricarboxylic acid (TCA) cycle were broadly upregulated, indicating active coupling between carbon oxidation and energy generation. In particular, transcripts encoding *korB* and *sucCD*, which are involved in 2-oxoglutarate conversion to succinyl-CoA and subsequent substrate-level phosphorylation [35], increased by 1.37- and 1.16-fold, respectively (Fig. S11), suggesting enhanced carbon flux through oxidative metabolism under low FA stress.

The coordination began to weaken at moderate FA levels (Phase III). Although photosynthetic reactions remained partially active, several TCA-associated genes (*acnA*, *icd*, *mdh*, and *korB*) exhibited consistent downward trends (Fig. S11), indicating progressive decoupling between carbon oxidation and energy recovery. Under the highest FA stress (25.0 mg/l, Phase IV), suppression of the TCA cycle activity became more evident, with *sucCD* transcripts declining by 27.1% (Fig. S11). This pattern reflected a broad suppression of oxidative carbon metabolism and ATP-generating capacity.

Concomitantly, genes associated with the glyoxylate cycle exhibited a distinct response pattern. The expression of key genes initially increased and then declined during Phases I–III, but partially rebounded in Phase IV, with transcripts of *aceA*, *aceB*, *pckA*, and *acs* increasing by 1.11-, 1.21-, 1.13-, and 1.05-fold, respectively (Fig. S12). This shift indicates a rerouting of carbon metabolism toward glyoxylate-dependent pathways that can conserve carbon skeletons and reduce NADH production [36]. Such a metabolic adjustment may help maintain cellular survival under severe FA stress, although the reduced generation of reducing equivalents could constrain electron supply for denitrification.

Collectively, increasing FA stress redirected the metabolic strategy of the OPG community from microbial signaling-mediated cooperation toward self-maintenance-oriented metabolism. The weakening of signaling-associated cross-feeding networks disrupted energy homeostasis and ultimately undermined cooperative nitrogen transformations, leading to the deterioration of nitrogen removal performance.

## Discussion

### FA stress reconfigures microbial signaling and regulatory networks in OPGs

Microbial signaling networks enable microorganisms to coordinate collective behavior by coupling signaling production, perception, and transcriptional regulation across populations [37]. Environmental stress can disrupt this coordination by altering signal abundance, regulatory activity, and the coupling among different signaling systems [38]. Although previous studies have largely focused on the responses of individual signaling molecules to environmental stresses [20], how complex microbial communities reorganize multiple signaling and regulatory pathways under chemical stress remains poorly understood. In this study, FA stress induced a threshold-dependent reconfiguration of signaling-associated regulation in OPGs. Rather than causing a simple monotonic inhibition, increasing FA exposure triggered a staged transition from DSF- and IAA-associated signaling under low FA exposure to enhanced AHL- and c-di-GMP-associated regulation under moderate FA stress, and finally to a broad transcriptional attenuation under severe FA stress. This pattern suggests that dynamic signaling reconfiguration represents an important adaptive strategy through which OPG communities respond to increasing FA stress.

Under low FA stress, DSF- and IAA-associated pathways were more transcriptionally active. DSF has been widely associated with stress sensing, biofilm development, and behavioral flexibility in bacterial communities [39]. In parallel, IAA can act as an interpopulation signaling molecule involved in bacterial-phototrophic interactions, metabolic activation, and community development [40]. The concurrent activation of DSF- and IAA-associated pathways may therefore support early-stage photogranule consolidation by promoting metabolic plasticity, resource assimilation, and cooperative interactions among community members [41]. This interpretation is consistent with the enhanced EPS production, increased pigment accumulation, and compact granule structure observed during Phases I and II (Fig. 1).

A clear regulatory transition occurred under moderate FA stress (5.0 mg/l; Phase III). At this stage, AHL-associated signaling and c-di-GMP-mediated regulation became more prominent. AHL-associated signaling enables fine-tuned coordination among phylogenetically related populations, particularly in density-dependent behaviors and community-level metabolic organization [42]. C-di-GMP is a central intracellular second messenger that promotes EPS synthesis and suppresses motility, thereby reinforcing biofilm architecture and structural protection [43]. The increased activity of AHL- and c-di-GMP-mediated pathways under moderate FA stress may therefore reflect a shift from growth-oriented cooperation toward stress-adaptive regulation, in which the community prioritized aggregation, structural protection, and persistence.

The coordinated response of these pathways was further supported by correlations among homologous and heterologous signaling systems (Fig. 2C and D). These associations suggest that OPGs did not rely on a single signaling route, but instead integrated multiple signaling and regulatory modules to synchronize community responses across different functional populations. Such cross-system coordination may increase community robustness by allowing microbial populations to adjust aggregation, resource exchange, and stress responses simultaneously. The genetic repertoire of signaling-associated pathways remained largely present across phases, whereas their transcriptional activity changed markedly. This indicates that FA primarily reshaped signaling networks at the regulatory activity level rather than by eliminating the underlying genetic potential.

Under severe FA exposure, adaptive coordination appeared to be overwhelmed. At 25.0 mg/l FA (Phase IV), the transcription of genes involved in signaling synthesis, perception, and regulation was broadly suppressed across the four signaling systems (Fig. 3B). This broad attenuation suggests that severe FA stress weakened the community’s ability to maintain coordinated signaling-associated regulation. As a result, the signaling network may have shifted from an adaptive regulatory layer that supported collective responses to a constrained system unable to sustain cross-system coordination. Taken together, these findings indicate that FA stress rewires microbial signaling networks in a threshold-dependent manner: moderate stress promotes adaptive network reconfiguration, whereas severe stress suppresses signaling-associated regulation and limits coordinated community responses.

### Signaling-mediated cooperation shifts metabolic optimization to structural reinforcement under FA stress

Metabolic cross-feeding is a fundamental mechanism underlying microbial community stability and resilience. Through the exchange of amino acids, vitamins, cofactors, and other shared metabolites, microorganisms can partition metabolic functions, reduce redundant metabolic investment, and maintain cooperative interactions that support community function [44]. Increasing evidence suggests that microbial signaling helps coordinate these exchanges, particularly when communities need to balance cellular growth, resource allocation, and stress resistance under fluctuating environmental conditions [45].

In the OPG system investigated, AHL- and DSF-mediated signaling pathways were closely associated with the organization of metabolic cross-feeding networks. Metatranscriptomic analysis revealed a clear functional partitioning among dominant MAGs, particularly in amino acid biosynthesis (Fig. S5). The uneven distribution of biosynthetic functions among dominant MAGs, together with the coupling between signaling activity and metabolic gene expression, suggests that signaling-mediated regulation helped coordinate metabolite production and exchange across populations [46]. Under low FA exposure, this coordination likely promoted cooperative growth, EPS accumulation, photogranule development, and efficient nitrogen transformation (Fig. 1). Thus, microbial signaling appeared to connect metabolite exchange with community-level structural and functional stability, allowing the consortium to optimize metabolic investment with concomitant maintenance of reactor performance.

As FA increased to a moderate level, signaling-mediated cooperation appeared to shift from metabolic optimization toward structural reinforcement. Enhanced AHL-associated activity and c-di-GMP-mediated intracellular regulation coincided with improved aggregation, partial recovery of EPS content, and maintenance of photogranule morphology. This response suggests an adaptive trade-off: under increasing FA pressure, the community maintained cooperative exchange of public goods and allocated more regulatory effort toward biofilm stability, aggregation, and stress protection [47]. In this sense, moderate FA stress did not immediately disrupt cooperation, but reshaped its ecological function from growth-oriented metabolic efficiency toward self-maintenance-oriented community protection [48].

Once FA stress exceeded the community’s capacity to sustain reciprocal exchange, the cooperative strategy became increasingly vulnerable. Specifically, metatranscriptomic evidence showed that several key populations, such as BAC2, significantly downregulated genes involved in the biosynthesis of public goods, including vitamins (e.g. VB1 and VB12), cofactors (e.g. MOCO), and key amino acids, in Phase IV (Fig. S7). This repression was accompanied by weakened metabolic cross-feeding among MAGs (Fig. S9), indicating disruption of interpopulation metabolite exchange networks. In parallel, signaling-associated regulation was broadly attenuated across AHL-, DSF-, IAA-, and c-di-GMP-related pathways (Fig. 3C), suggesting a loss of transcriptional coordination required to sustain cooperative metabolism. Concomitantly, intracellular ATP levels reached their lowest point under severe FA stress (Fig. S3), reflecting insufficient energy availability to support energetically expensive signal production and metabolite biosynthesis. As a result, microbial populations may have increasingly relied on autonomous biosynthetic pathways or alternative uptake strategies, as further evidenced by carbon-conserving metabolic reprogramming (Fig. S12). This shift likely aggravated the cellular energy demand–supply imbalance, weakened metabolite availability, and ultimately contributed to reduced reactor performance (Fig. 1I).

### Energy imbalance and metabolic reallocation under extreme FA stress

Intracellular ATP is the central energetic currency through which microorganisms balance energy acquisition and expenditure [49]. Previous studies have mainly emphasized impaired ATP generation or the inhibition of specific microbial guilds under FA stress [50]. Here, our results further demonstrate that FA stress disrupted cellular energy homeostasis through simultaneous suppression of energy generation and escalation of energy demand.

During Phases I and II, the OPGs maintained stable physicochemical properties, active signaling-mediated cooperation, and relatively high intracellular ATP levels, enabling the community to buffer low FA exposure. However, this balance became less stable during Phase III. The increased energetic demand required to maintain intracellular pH and ion gradients may have progressively diverted ATP utilization away from cooperative functions. As a result, microbial signaling networks that previously supported efficient nitrogen removal began to undergo restructuring and partial downregulation. This phase therefore represented a critical energetic transition, in which transcriptional investment appeared to shift from cooperative functions, including microbial signaling, amino acid, and cofactor biosynthesis for cross-feeding, and nitrogen metabolism, toward essential survival processes, including K^+^/Na^+^ ion homeostasis mediated by Trk, Kup, and NhaBC systems, pH regulation, and macromolecular repair.

At the highest FA concentration (25.0 mg/l, Phase IV), the microbial community experienced severe bioenergetic limitation. The decline in photosynthetic and respiratory energy-generation pathways, together with reduced ATP synthase expression and lower intracellular ATP levels, indicated impaired bioenergetic capacity (Fig. 5). Under such energy-limited conditions, microorganisms generally prioritize essential physiological processes, including maintenance of transmembrane ion gradients, repair of damaged macromolecules, and basal protein turnover, over energetically costly cooperative activities that generate delayed or community-level benefits [51]. Consequently, cellular metabolism may shift toward a low-activity state that minimizes energy expenditure, concurrently preserving basic viability [52].

Signaling-mediated metabolic cross-feeding is beneficial at the community level, but it requires sustained energetic investment in signal production, transcriptional regulation, and metabolite biosynthesis [53]. Its evolutionary advantage therefore depends on a positive cost-benefit balance, in which cooperative returns offset the energetic costs of coordinated metabolic activity [54]. Under severe FA stress, the reduced energy supply likely made this cooperative strategy difficult to maintain. This interpretation is consistent with the broad attenuation of signaling-associated pathways, reduced transcription of shared metabolite biosynthesis genes, and impaired nitrogen transformation observed in Phase IV (Figs S9 and 3C). The upregulation of glyoxylate shunt-related genes suggests a shift toward carbon-conserving metabolism (Fig. S12). By bypassing the decarboxylation steps of the TCA cycle, the glyoxylate shunt can conserve carbon skeletons for biosynthesis, although it may reduce the generation of reducing equivalents and ATP compared with complete oxidative metabolism [55]. Collectively, these metabolic adjustments indicate a community-wide transition in OPGs from cooperation-driven metabolic optimization under low FA exposure toward self-maintenance-oriented energy conservation under severe FA stress.

### Implications for microbial social organization under FA stress

Microbial signaling enables communities to coordinate collective behaviors and function as integrated ecological units rather than as isolated populations [56]. In this study, microbial signaling emerged as a regulatory axis linking communication, metabolic cooperation, and cellular energy allocation in OPGs under FA stress. Unlike previous studies that have mainly emphasized the cytotoxic effects of FA or its selective inhibition of ammonia- and nitrite-oxidizing bacteria [57], our multi-omics analyses indicate that FA stress can also reshape the cooperative organization and energy economy of microbial communities.

By integrating signaling dynamics, metabolic cross-feeding networks, and cellular energy allocation strategies, this study provides a mechanistic framework in which increasing FA stress progressively reconfigures communication-mediated cooperation, alters resource allocation, and ultimately constrains nitrogen removal in OPGs. These findings extend the understanding of FA stress from direct physiological inhibition to a community-level ecological process involving the reorganization of microbial signaling, cooperation, and energy use. They also suggest that the resilience of engineered microbial consortia depends not only on the presence of functional genes or metabolic capacity, but also on the integrity of signal-mediated cooperative networks under energetic stress. In this context, microbial signaling may serve as both a regulatory mechanism and a potential ecological indicator of whether a community can maintain cooperative organization when confronted with chemical disturbance.

These interpretations should be considered in light of the sequential FA-loading regime and biomass retention conditions of the reactor. Throughout the experiment, mixing speed, cycle configuration, illumination, hydraulic retention time, and sampling volume were kept constant across phases, and no intentional sludge wasting was performed. Therefore, changes in granule size and biomass were not attributable to deliberate shifts in mixing or wasting regimes. Nevertheless, because operation time, FA exposure, microbial acclimation, and potential effluent biomass loss are inherently linked in a sequential reactor system, these factors cannot be completely separated from FA effects. We therefore interpret the observed responses as FA-associated, concentration-dependent changes occurring under a sequential loading regime, rather than as fully isolated effects of FA concentration alone. This interpretation is supported by the ANCOVA results, non-monotonic phase-specific responses, and coordinated changes in signaling regulation, energy metabolism, photogranule structure, and nitrogen removal performance.

More broadly, the dynamic coupling and decoupling of microbial signaling, metabolic cooperation, and energy homeostasis observed here may reflect an ecological pattern underlying microbial adaptation to chemical stress. Recognizing these interactions provides a framework for monitoring and managing the stability of phototrophic wastewater treatment systems under FA disturbance, particularly by maintaining the cooperative networks that support nitrogen removal and granular integrity.

## Conclusion

The study demonstrates that FA stress reshapes microbial signaling, metabolic cooperation, and cellular energy allocation in OPGs in a concentration-dependent manner. Under low FA exposure (≤ 1.0 mg/l), DSF- and IAA-associated activities were linked to active metabolic exchange, photogranule consolidation, and stable nitrogen removal. At moderate FA exposure (5.0 mg/l), the community shifted toward enhanced AHL-associated signaling and c-di-GMP-mediated regulation, coinciding with structural reinforcement and stress adaptation. In contrast, severe FA stress (25.0 mg/l) broadly attenuated signaling-associated pathways, weakened metabolic cross-feeding, impaired energy generation, and promoted a shift toward self-maintenance-oriented carbon metabolism, as reflected by the upregulation of glyoxylate shunt-related genes. These findings suggest that FA stress impairs OPG performance not only through direct physiological inhibition, but also by disrupting signaling-mediated cooperation and cellular energy homeostasis. Overall, this work advances an energy-allocation perspective for understanding microbial social stability under chemical stress and provides ecological guidance for developing more FA-resilient phototrophic wastewater treatment systems.

## Supplementary Material

Revised_Supplementary_Material_wrag225

## Data Availability

The metagenomic and metatranscriptomic raw sequencing data generated in this study have been deposited in the NCBI Sequence Read Archive (SRA) database under accession code PRJNA1403730. All other source data supporting the findings of this study are available from the corresponding author upon reasonable request.
