# Supplementary material for: Free ammonia stress rewires microbial signaling networks and constrains metabolic cooperation in oxygenic photogranules
Source: ISME J. 2026 Sep 2;20(1):wrag225. doi: 10.1093/ismejo/wrag225 (PMC13637823; doi:10.1093/ismejo/wrag225)
Supplement: Supplementary file 1 [file revised_supplementary_material_wrag225.docx]

*Supplementary Material*

**Free Ammonia Stress Rewires Microbial Signaling Networks and Constrains Metabolic Cooperation in Oxygenic Photogranules**

**Bing Zhang^a, b^, Xinya Xu^a, b^, Ming Zhang^a, b^,** **Bowen Qi^a, b^, Hua Ma ^a, b^, Peng Yan^a, b^, Piet N.L. Lens^c^, Wenxin Shi^a, b,[1]*^**

*^a^ Key Laboratory of the Three Gorges Reservoir Region’s Eco-Environment, Ministry of Education, Chongqing University, 400045 Chongqing, China*

*^b^ College of Environment and Ecology, Chongqing University, 400044 Chongqing, China*

*^c^ IHE Delft Institute for Water Education, Westvest 7, 2601 DA Delft, the Netherlands*

** Corresponding author: Wenxin Shi, College of Environment and Ecology, No.83 Shabei Street, Shapingba District, Chongqing University, Chongqing 400045, China. E-mail: swx@hit.edu.cn*

**Text captions**

**Text S1.** Cultivation procedure of the seed photogranules.

**Text S2.** Composition of the synthetic wastewater.

**Text S3.** Extraction protocol and component determination of EPS.

**Text S4.** Extraction and analysis of signaling molecules.

**Text S5.** Protocols for determining the SAOR, SNOR, SNIRR, SNRR, and ETSA.

**Figure captions**

**Fig. S1.** Photomicrographs of OPGs obtained at different operational phases. (A) day 0, (B) day 20, (C) day 40, (D) day 80, and (E) day 120. Scale bar = 100 μm.

**Fig. S2.** Scanning electron microscope (SEM) images of OPGs obtained at different operational phases. (A) Phase I (0.1 mg/L FA), (B) Phase II (1 mg/L FA), (C) Phase III (5 mg/L FA), and (D) Phase IV (25 mg/L FA).

**Fig. S3.** Variation of ATP content in four phases. Phase I (0.1 mg/L FA), Phase II (1 mg/L FA), Phase III (5 mg/L FA), and Phase IV (25 mg/L FA).

**Fig. S4.** Correlations between the concentration of signaling molecules and FA levels.

**Fig. S5.** Relative gene expression involved in amino acid biosynthetic pathways encoded by each MAG. (A) Phase I vs. Phase II, (B) Phase II vs. Phase III, and (C) Phase III vs. Phase IV. Asterisks (*) indicate the significant differences with Phase I, Phase II, and Phase III: *, *P* < 0.05; **, *P* < 0.01; ***, *P* < 0.001.

**Fig. S6.** Relative gene expression of amino acid transporters and peptidases encoded by each MAG: (A) Phase I vs. Phase II, (B) Phase II vs. Phase III, and (C) Phase III vs. Phase IV. Asterisks (*) indicate the significant differences with Phase I, Phase II, and Phase III: *, *P* < 0.05; **, *P* < 0.01; ***, *P* < 0.001.

**Fig. S7.** Relative gene expression of cofactor synthesis pathways encoded by each MAG. (A) Phase I vs. Phase II, (B) Phase II vs. Phase III, and (C) Phase III vs. Phase IV. Asterisks (*) indicate the significant differences with Phase I, Phase II, and (c) Phase III: *, *P* < 0.05; **, *P* < 0.01; ***, *P* < 0.001.

**Fig. S8.** Binding sites and relative expression levels of the Clp regulon and the LuxR regulon in the promoters of amino acid biosynthesis in PSE1, PSE3, PSE4, PSE5, CYA1, and CYA2. Asterisks (*) indicate the significant differences with Phase I: *, *P* < 0.05; **, *P* < 0.01; ***, *P* < 0.001.

**Fig. S9.** Schematic illustration of the nitrogen removal mechanism.

**Fig. S10.** Relative expression levels of kup, trk, kef, and Nhabc in OPGs collected from Phases I-IV.

**Fig. S11.** Differential expression of tricarboxylic acid (TCA) cycle genes in OPGs across different operational phases. (A) Schematic illustration of the TCA cycle; (B) Differential expression profiles of TCA cycle genes in OPGs across different FA exposure phases (Phase I vs. II, Phase II vs. III, and Phase III vs. IV).

**Fig. S12.** Differential expression profiles of glyoxylate cycle genes in OPGs across different FA exposure phases (Phase I vs. II, Phase II vs. III, and Phase III vs. IV).

**Table captions**

**Table S1.** Genome statistics of MAGs recovered from OPG systems.

**Table S2.** ANCOVA results for the effects of FA phase and operation time on TN removal rate.

**Table S3.** ANCOVA results for the effects of FA phase and operation time on ammonium removal rate.

**Table S4.** ANCOVA results for the effects of FA phase and operation time on average particle size.

**Table S5.** ANCOVA results for the effects of FA phase and operation time on chlorophyll concentration.

**Table S6.** ANCOVA results for the effects of FA phase and operation time on phycobilin concentration.

**Table S7.** ANCOVA results for the effects of FA phase and operation time on mixed liquor suspended solids.

**Table S8.** ANCOVA results for the effects of FA phase and operation time on SVI_30_.

**Texts**

**Text S1**. Cultivation procedure of the seed photogranules.

The seed photogranules were generated by hydrostatic incubation of activated sludge under continuous illumination following a previously published method [1]. Activated sludge was collected from the aeration basin of a municipal wastewater treatment plant (WWTP) in Chongqing, China. Aliquots (5 mL) of the sludge were inoculated into sterile 10 mL glass vials, which were subsequently sealed with plastic caps. The vials were incubated statically, without mixing or agitation, at a constant temperature of 23 ± 3 °C and exposed to continuous illumination provided by fluorescent lamps. The photosynthetically active radiation (PAR) intensity measured at the top of the vials was approximately 150 µmol/m^2^·s. After three weeks of hydrostatic incubation, spherical photogranules with diameters ranging from 5 to 15 mm were consistently observed in the vials and used as seed photogranules for this study.

**Text S2.** Composition of the synthetic wastewater.

Synthetic wastewater was prepared with the following composition. For each 5 L of wastewater, sodium acetate (1.92 g) was added as the carbon source, corresponding to 300 mg/L COD. Phosphorus was supplied as KH_2_PO_4_ (0.13 g; 5 mg/L PO_4_^3-^-P). Calcium, magnesium, and iron were provided as CaCl_2_ (0.05 g; 30 mg/L Ca^2+^), MgSO_4_·7H_2_O (0.25 g; 50 mg/L Mg^2+^), and FeSO_4_·7H_2_O (0.05 g; 10 mg/L Fe^2+^), respectively. In addition, 1.0 mL/L of a trace element solution was supplemented. The trace element solution contained (in mg/L): ZnSO_4_·7H_2_O 120, Na_2_Mo_7_O_24_·2H_2_O 60, CoCl_2_·6H_2_O 60, CuSO_4_·5H_2_O 30, MnCl_2_·4H_2_O 100, H_3_BO_3_ 100, AlCl_3_ 50, NiCl_2_ 40 [2].

Ammonium was supplied as NH_4_Cl. For each 5 L of wastewater, NH_4_Cl was dosed at 0.382 g, 0.955 g, 1.911 g, and 2.291 g in Phases I-IV, respectively. The pH of the aqueous phase was adjusted by adding NaHCO₃ at amounts of 0.1 g, 0.25 g, 0.5 g, and 0.75 g in Phases I-IV, respectively.

**Text S3.** Extraction protocol and component determination of EPS.

Extracellular polymeric substances (EPS) were extracted using a modified thermal extraction method [3], which separated EPS into loosely bound EPS (LB-EPS) and tightly bound EPS (TB-EPS). The concentrations of proteins (PN) and polysaccharides (PS) in the EPS were determined by the Lowry-Folin method and phenol-sulfuric acid method, respectively [4].

**Text S4.** Extraction and analyses of signaling molecules.

(1) Extraction of AHL, DSF, and IAA

Acyl-homoserine lactones (AHL), diffusible signal factors (DSF) and indole-3-acetic acid (IAA) were extracted following previously reported methods with minor modifications [5,6]. Briefly, 200 mL of reactor effluent was collected and filtered through a glass fiber membrane filter. The filtrate was concentrated using solid-phase extraction (SPE). The SPE cartridge (Oasis HLB, Waters, USA) was preconditioned with 5 mL methanol followed by 5 mL ultrapure water. The retained compounds were eluted with 5 mL methanol, evaporated to dryness under a gentle nitrogen stream, and reconstituted in 1 mL methanol. To account for potential variations in biomass across samples, signal abundances were normalized to MLSS. The extracts were filtered prior to analysis by liquid chromatography-tandem mass spectrometry (LC-MS/MS). Detailed LC-MS/MS conditions are provided below.

(2) Extraction of c-di-GMP

The extraction method of bis-(3'-5')-cyclic dimeric guanosine monophosphate (c-di-GMP) followed the protocol described by Guo et al. [7]. After the aeration period, sludge samples (25 mL) were collected and centrifuged (8000 g, 10 min, 4℃) to remove the supernatant. The pellets were resuspended in ultrapure water (5 mL) and subjected to ultrasonication (3 s/3 s, 200 W, 20 min). Subsequently, a mixture of methanol and acetonitrile (1:1, v/v; 20 mL) was added, followed by brief vortexing (5 s). Finally, the treated samples were refrigerated (-20℃) for protein precipitation over a period of 4 h before being centrifuged again (11000 g, 20 min, 4℃), filtered through 0.22 μm PTFE filters and analyzed using LC-MS/MS. The detailed information of LC-MS/MS conditions are provided below. Signaling abundance was normalized to MLSS to account for changes in biomass, ensuring that the observed decline reflects transcriptional and signaling responses rather than reduced cell density.

(3) LC-MS/MS conditions

LC-MS/MS analysis was performed using an Waters C18 column (Waters Corp., Milford, MA) operated in positive electrospray ionization (ESI^+^) mode. Quantification was conducted using multiple reaction monitoring (MRM). The mobile phases for AHL analysis consisted of water with 0.1% formic acid (solvent A) and acetonitrile with 0.1% formic acid methanol (solvent B). The mobile phases of DSF analysis comprised a 5.0% buffer solution (solvent A) and a mixture of water containing 5.0% buffer solution, 55.0% acetonitrile, and 40.0% isopropanol (solvent B). For c-di-GMP, the mobile phases were composed of water containing 0.05% aqueous ammonia (solvent A) and acetonitrile (solvent B).

**Text S5.** Protocols for determining SAOR, SNOR, SNIRR, SNRR, and ETSA.

Specific nitrogen transformation rates were determined following the method of Qin et al. [8] with minor modifications.

For determination of specific ammonia oxidation rate (SAOR), 20 mL of oxygenic photogranules (OPGs) was mixed with 70 mL of culture medium containing 14 mg/L Na_2_HPO_4_ and 120 mg/L NH_4_Cl in a 150 mL conical flask. The suspension was continuously aerated and magnetically stirred to maintain aerobic conditions. Samples were collected at 30 min intervals for NH_4_^+^-N analysis. After completion of the experiment, the MLSS concentration was measured, and SAOR was calculated as the unit mass ammonia oxidation rate per unit sludge mass and time.

For determination of the specific nitrite oxidation rate (SNOR) and specific nitrite reduction rate (SNIRR), a similar procedure was employed, with the culture medium comprising 14 mg/L Na_2_HPO_4_ and 147 mg/L NaNO_2_. Aerobic and anaerobic conditions were established to determine specific nitrite oxidation rates (SNOR) and specific nitrite reduction rates (SNIRR), respectively. Nitrite concentrations were monitored at 30-min intervals, with the resulting rates normalized to the MLSS concentration.

To determine the specific nitrate reduction rate (SNRR), a culture medium containing 14 mg/L Na_2_HPO_4_ and 303 mg/L NaNO_3_ was used under anaerobic conditions. Nitrate concentrations were monitored at 30-min intervals, and the SNRR was calculated and normalized to the MLSS concentration.

The electron transport system activity (ETSA) was tested by reducing 2-(p-iodophenyl)-3-(pnitrophenyl)-5-phenyl tetrazolium chloride (INTC) to formazan. The absorbance of orange-like formazan was immediately detected at 490 nm against a solvent blank. The ETSA was calculated according to the following equation [9]:

$$\text{ }\text{ETSA }\text{(}\text{μg }\text{O}_{\text{2}}\text{ }\text{g}^{\text{-1}}\text{ }\text{protein }\text{h}^{\text{-1}}\text{ }\text{)}\text{ }\text{= }\text{16 }\text{× }\text{ABS}_{\text{490}}\text{ }\text{× }\text{V}\text{/}\text{(}\text{15}\text{.}\text{9 }\text{× }\text{V}_{\text{0}}\text{ }\text{× }\text{t}\text{ }\text{× }\text{M}\text{)}\text{ (1)}$$

where ABS_490_ is the absorbance of the sample; 15.9 is the absorptivity of INT-formazan; *V_0_* and *V* (mL) are the volumes of initial suspension and methanol, respectively; *t* (h) is the incubation time; 16 is the factor for transformation of INT-formazan to O_2_; and *M* (mg protein g^− 1^ MLSS) is the protein content per gram of MLSS.

**Figures**

**Fig. S1.** Photomicrographs of OPGs obtained at different operational phases. (A) day 0, (B) day 20, (C) day 40, (D) day 80, and (E) day 120. Scale bar = 100 μm.

**Fig. S2.** Scanning electron microscope (SEM) images of OPGs obtained at different operational phases. (A) Phase I (0.1 mg/L FA), (B) Phase II (1 mg/L FA), (C) Phase III (5 mg/L FA), and (D) Phase IV (25 mg/L FA).

**Fig. S3.** Variation of ATP content in four phases. Phase I (0.1 mg/L FA), Phase II (1 mg/L FA), Phase III (5 mg/L FA), and Phase IV (25 mg/L FA).

**Fig. S4.** Correlations between the concentration of signaling molecules and FA levels.

**Fig. S5.** Relative gene expression of amino acid biosynthetic pathways encoded by each MAG. (A) Phase I vs. Phase II, (B) Phase II vs. Phase III, and (C) Phase III vs. Phase IV. Asterisks (*) indicate the significant differences with Phase I, Phase II, and Phase III: *, *P* < 0.05; **, *P* < 0.01; ***, *P* < 0.001.

**Fig. S6.** Relative gene expression of amino acid transporters and peptidases encoded by each MAG: Phase I vs. Phase II, Phase II vs. Phase III, and Phase III vs. Phase IV. Asterisks (*) indicate the significant differences with Phase I, Phase II, and Phase III: *, *P* < 0.05; **, *P* < 0.01; ***, *P* < 0.001.

**Fig. S7.** Relative gene expression of cofactor synthesis pathways encoded by each MAG. (A) Phase I vs. Phase II, (B) Phase II vs. Phase III, and (C) Phase III vs. Phase IV. Asterisks (*) indicate the significant differences with Phase I, Phase II, and Phase III: *, *P* < 0.05; **, *P* < 0.01; ***, *P* < 0.001.

**Fig. S8.** Binding sites and relative expression levels of the Clp regulon and the LuxR regulon in the promoters of amino acid biosynthesis in PSE1, PSE3, PSE4, PSE5, CYA1, and CYA2. Asterisks (*) indicate the significant differences with Phase I: *, *P* < 0.05; **, *P* < 0.01; ***, *P* < 0.001.

**Fig. S9.** Schematic illustration of the nitrogen removal mechanism.

**Fig. S10.** Relative expression levels of kup, trk, kef, and Nhabc in OPGs collected from Phases I-IV.

**Fig. S11.** Differential expression of tricarboxylic acid (TCA) cycle genes in OPGs across different operational phases. (A) Schematic illustration of the TCA cycle; (B) Differential expression profiles of TCA cycle genes in OPGs across different operational phases (Phase I vs. II, Phase II vs. III, and Phase III vs. IV).

**Fig. S12.** Differential expression profiles of glyoxylate cycle genes in OPGs across different operational phases (Phase I vs. II, Phase II vs. III, and Phase III vs. IV).

# Tables

## Table S1. Genome statistics of MAGs recovered from OPG systems.

| **MAG ID** | **Taxonomy** | **Completeness**  **(%)** | **Contamination**  **(%)** | **Genome size**  **(bp)** | **N50 length**  **(bp)** |
| --- | --- | --- | --- | --- | --- |
| PSE1 | Bacteria; Pseudomonadota;  Alphaproteobacteria; Sphingomonadales; Sphingomonadaceae; Novosphingobium; Novosphingobium ginsenosidimutans | 65.8 | 0.3 | 1921382 | 4102 |
| PSE2 | Bacteria; Pseudomonadota; Alphaproteobacteria; Sphingomonadales; Sphingomonadaceae; Sphingorhabdus_B; Unclassified | 100.0 | 0.2 | 2986077 | 235639 |
| PSE3 | Bacteria; Pseudomonadota; Alphaproteobacteria; Sphingomonadales; Sphingomonadaceae; 34-65-8; Unclassified | 95.0 | 1.2 | 3015046 | 11827 |
| PSE4 | Bacteria; Pseudomonadota; Alphaproteobacteria; Sphingomonadales; Sphingomonadaceae; Sphingopyxis; Sphingopyxis sp020160755 | 94.4 | 2.7 | 2724648 | 2989 |
| PSE5 | Bacteria; Pseudomonadota; Alphaproteobacteria; Rhodobacterales; Rhodobacteraceae; Albidovulum; Albidovulum sp020637675 | 65.8 | 2.2 | 2724648 | 2989 |
| BAC1 | Bacteria; Bacteroidota; Bacteroidia; Cytophagales; Spirosomaceae; Runella; Unclassified | 90.9 | 1.4 | 6916839 | 12050 |
| BAC2 | Bacteria; Bacteroidota; Bacteroidia; Cytophagales; Spirosomaceae; Runella; Unclassified | 86.4 | 0.1 | 5421181 | 90221 |
| ACI | Bacteria; Actinomycetota; Acidimicrobiia; Acidimicrobiales; Ilumatobacteraceae; Ilumatobacter; Unclassified | 81.8 | 5.2 | 5243057 | 5493 |
| CYA1 | Bacteria; Cyanobacteriota; Cyanobacteriia; Elainellales; Elainellaceae; Elainella; Unclassified | 67.9 | 0.6 | 3706203 | 3130 |
| CYA2 | Bacteria; Cyanobacteriota; Cyanobacteriia; Elainellales; Elainellaceae; FACHB-8; Unclassified | 92.8 | 2.6 | 8307931 | 7726 |

## Table S2. ANCOVA results for the effects of FA phase and operation time on TN removal rate.

| Source | Type III Sum of Squares | df | Mean Square | *F* | Significance | Partial Eta Squared |
| --- | --- | --- | --- | --- | --- | --- |
| Time | 0.422 | 1 | 0.422 | 0.3 | 0.612 | 0.018 |
| FA | 589.028 | 3 | 196.343 | 125.0 | 0.000 | 0.962 |

## Table S3. ANCOVA results for the effects of FA phase and operation time on ammonium removal rate.

| Source | Type III Sum of Squares | df | Mean Square | *F* | Significance | Partial Eta Squared |
| --- | --- | --- | --- | --- | --- | --- |
| Time | 0.051 | 1 | 0.051 | 0.2 | 0.677 | 0.012 |
| FA | 418.078 | 3 | 139.359 | 491.5 | 0.000 | 0.990 |

## Table S4. ANCOVA results for the effects of FA phase and operation time on average particle size.

| Source | Type III Sum of Squares | df | Mean Square | *F* | Significance | Partial Eta Squared |
| --- | --- | --- | --- | --- | --- | --- |
| Time | 288.315 | 1 | 288.315 | 3.8 | 0.069 | 0.204 |
| FA | 1562.866 | 3 | 520.955 | 6.9 | 0.004 | 0.581 |

## Table S5. ANCOVA results for the effects of FA phase and operation time on chlorophyll concentration.

| Source | Type III Sum of Squares | df | Mean Square | *F* | Significance | Partial Eta Squared |
| --- | --- | --- | --- | --- | --- | --- |
| Time | 20.038 | 1 | 20.038 | 2.1 | 0.167 | 0.204 |
| FA | 310.670 | 3 | 103.557 | 10.8 | 0.001 | 0.655 |

## Table S6. ANCOVA results for the effects of FA phase and operation time on phycobilin concentration.

| Source | Type III Sum of Squares | df | Mean Square | *F* | Significance | Partial Eta Squared |
| --- | --- | --- | --- | --- | --- | --- |
| Time | 2.244E^-5^ | 1 | 2.244E^-5^ | 1.4 | 0.249 | 0.077 |
| FA | 0.001 | 3 | 0.000 | 26.6 | 0.000 | 0.825 |

## Table S7. ANCOVA results for the effects of FA phase and operation time on mixed liquor suspended solids.

| Source | Type III Sum of Squares | df | Mean Square | *F* | Significance | Partial Eta Squared |
| --- | --- | --- | --- | --- | --- | --- |
| Time | 302760.000 | 1 | 302760.000 | 4.4 | 0.054 | 0.226 |
| FA | 2471643.683 | 3 | 823881.228 | 11.9 | 0.001 | 0.704 |

## Table S8. ANCOVA results for the effects of FA phase and operation time on SVI_30_.

| Source | Type III Sum of Squares | df | Mean Square | *F* | Significance | Partial Eta Squared |
| --- | --- | --- | --- | --- | --- | --- |
| Time | 20.693 | 1 | 20.693 | 4.1 | 0.060 | 0.217 |
| FA | 283.152 | 3 | 94.384 | 18.9 | 0.001 | 0.791 |

**REFERENCES**

1. Shi W, An Y, Cui F. et al. Growth progression driven by c-di-GMP in photogranules under hydrostatic conditions: polysaccharides biosynthesis and microbial interactions. Chem Eng J 2025;**524**:169476. <https://doi.org/10.1016/j.cej.2025.169476>
2. Shi W, Yang X, Huang S. et al. Micro(nano)plastic size and concentration co-differentiate the treatment performance and toxicity mechanism in aerobic granular sludge systems. Chem Eng J 2023;**457**:147619. <https://doi.org/10.1016/j.cej.2022.141212>
3. Guo Y, Zhang B, Feng S. et al. Unveiling significance of Ca^2+^ ion for start-up of aerobic granular sludge reactor by distinguishing its effects on physicochemical property and bioactivity of sludge. Environ Res 2022;**212**:113299. <https://doi.org/10.1016/j.envres.2022.113299>
4. Peterson GL. A simplification of the protein assay method of Lowry et al. which is more generally applicable. Anal Biochem 1977;**83**:346-356. https://doi.org/10.1016/0003-2697(77)90043-4
5. Pan J, Zhou J, Tang X. et al. Bacterial communication coordinated behaviors of whole communities to cope with environmental changes. Environ Sci Technol 2023;**57**:4253-4265. <https://doi.org/10.1021/acs.est.2c05780>
6. Xu F, Yu Y, Guan B. et al. Germin-like protein 1 interacts with proteasome regulator 1 to regulate auxin signaling by controlling Aux/IAA homeostasis. Cell Rep 2025;**44**:116056. <https://doi.org/10.1016/j.celrep.2025.116056>
7. Guo Y, Liu S, Tang X. et al. Insight into c-di-GMP regulation in anammox aggregation in response to alternating feed loadings. Environ Sci Technol 2017;**51**:9155-9164. <https://doi.org/10.1021/acs.est.6b06396>
8. Qin R, Su C, Liu W. et al. Effects of exposure to polyether sulfone microplastic on the nitrifying process and microbial community structure in aerobic granular sludge. Bioresour Technol 2020;**302**:122827. <https://doi.org/10.1016/j.biortech.2020.122827>
9. Shi W, Yang X, Huang S. et al. Comprehensive analysis reveals the differentiated influential mechanism of degradable poly (ε-caprolactone) and polybutylene succinate microplastics on nitrogen transformation in aerobic granular sludge systems. Chem Eng J 2024;**479**:147619. <https://doi.org/10.1016/j.cej.2023.147619>
